# Risk assessment of paternal depression in relation to partner delivery during COVID-19 pandemic in Wuhan, China

**DOI:** 10.1186/s12888-021-03325-9

**Published:** 2021-07-02

**Authors:** Guo-qiang Sun, Qi Wang, Shan-shan Wang, Yao Cheng

**Affiliations:** 1 Obstetric Department, Maternal and Child Health Hospital of Hubei Province, Huazhong University of Science and Technology, Wuluo Road No.745, Hongshan District, Wuhan, 430070 China; 2 Department of Epidemiology and Biostatistics, School of Public Health, Tongji Medical College, Huazhong University of Science and Technology, Hangkong Road No.13, Qiaokou District, Wuhan, 430030 China

**Keywords:** Paternal perinatal depression, COVID-19, Traffic restriction, Family function, Risk factors

## Abstract

**Background:**

The COVID-19 pandemic and relevant prevention and control measures may affect the mental health and induce depressive symptoms in fathers with concurrent partner delivery exposure. This study aims to investigate the prevalence of depression in fathers with simultaneous exposure to COVID-19 pandemic and the effects of family functions on paternal perinatal depression (PPD) risk.

**Methods:**

A cross-sectional study was conducted among the perinatal fathers recruited in a large tertiary hospital in Wuhan across the whole pandemic period from 31 December 2019 to 11 April 2020. Edinburgh Postnatal Depression Scale (EPDS) and APGAR family function scale were used to evaluate PPD and family function, respectively. Chi-square test and multivariable-adjusted logistic regression model were applied for data analysis.

**Results:**

Among the 1187 participants, the prevalence of PPD was 13.82% throughout the COVID-19 pandemic. Compared with that in the time period before the announcement of human-to-human transmission on 19 January 2020, the depression risk was significantly lower during the traffic restriction (OR = 0.54, 95% CI: 0.34, 0.86) and public transportation reopening periods (OR = 0.29, 95% CI: 0.14, 0.59). Poor/fair family functions was associated with elevated depression risk (OR = 2.93, 95% CI: 1.90, 4.52). Individuals reporting a low family income and smoking had high depression risks.

**Conclusions:**

A declined risk of PPD was observed over the traffic restriction period of the COVID-19 pandemic. An improved family function may help alleviate the risk of PPD during the pandemic. Health authorities are recommended to formulate targeted prevention and control strategies to handle PPD.

**Supplementary Information:**

The online version contains supplementary material available at 10.1186/s12888-021-03325-9.

## Background

Parent mental health status is important in offspring development [1, 2]. Although maternal perinatal depression is widely studied, research on paternal perinatal depression (PPD) is inadequate. This condition has become an international public health concern because it may affect the well-being of individuals, their partners and offspring. PPD affects maternal depression [3] and contributes to increased risks of physical [4, 5], behavioural [6], and emotional problems among offspring [7, 8].

Coronavirus Disease 2019 (COVID-19) was first reported in December 2019 in Wuhan, Hubei province, China and has since spread worldwide. In addition to the direct physical health impairment caused by viral infections, the ongoing COVID-19 pandemic greatly threatens the mental health of pandemic-exposed individuals. The Chinese government promulgated *the Guidance Manual on the Prevention and Control of Novel Coronavirus-Infection Pneumonia in the Community* issued by the national government in response to the COVID-19 pandemic [9]. Hospitalized patients and their caregiver-companions are vulnerable to nosocomial COVID-19 infection, which may bring mental pressure on fathers in addition to the birth of new babies.

Home quarantine is implemented as an important measure to reduce human-to-human transmission in the COVID-19 control; however, this policy may also increase the role of family function among home-staying residents. Family function is an important reflection of social support. Insufficient social support during pregnancy could result in an increased risk of joint postnatal depression in mothers and fathers [10]. A systematic review reported the association between social support and PPD [11]. However, only a few studies focused on the prevalence of PPD and the role of family function during the COVID-19 pandemic.

To date, the prevalence and influential factors of PPD during the COVID-19 pandemic have never been addressed. This study aims to investigate the prevalence of PPD among the fathers exposed to the COVID-19 pandemic and to determine relevant risk factors. The findings provide new knowledge for the global prevention and control of PPD in the COVID-19 pandemic.

## Methods

### Research design and data collection

This study adopted a cross-sectional design and was conducted at a maternity care hospital located in Wuhan city, a tertiary hospital that is the largest maternal and childcare centre in the Hubei province. Data on family function, demographics and health behaviour factors were collected. Participants included men whose partners were hospitalized in their perinatal period (gestational age > 28 weeks and within 7 days after delivery) for delivery. Trained doctors and nurses introduced this survey to eligible individuals and obtained their oral agreement of participation from 31 December 2019 to 11 April 2020. A two-dimensional barcode of Questionnaire star (https://www.wjx.cn/) was provided to the participants and they could use their cellphone to fill in the survey in their spare time. Given that the nucleic acid detection method for COVID-19 was not fully developed in the early period of the pandemic, all participants were assumed to be not infected by COVID-19 when they had no clinical symptoms of COVID-19 including fever, respiratory symptoms and abnormal chest CT and had no history of direct contact with patients with COVID-19.

### Exposure measurements

PPD status was used as a dependent variable and assessed using the Chinese version of Edinburgh Postnatal Depression Scale (EPDS), which was compiled by Cox et al. in 1987 and is suitable for depression assessment because of its good reliability and validity [12]. The Chinese version of EPDS has been validated for paternal and maternal depressive symptoms [13, 14] and has been wildly used for fathers who may suffer depressive symptoms due to their partners’ antenatal and postnatal status [15, 16]. The scale consists of 10 items, each rated on a four-point scale ranging 0–3 by severity. A summary score of 10 points was used as a cut-off for PPD, which was reported as having a sensitivity of 82% and a specificity of 86% for Chinese fathers [17].

The pandemic time was divided into the following four periods to evaluate the effects of the COVID-19 pandemic on participants’ depression: (i) 1st period before the official announcement of human-to-human transmission by the National Health Commission of China on 19 January 2020 (31 December 2019 to 18 January 2020); (ii) 2nd period of thereafter to the day when traffic restrictions started (19–23 January 2020); (iii) 3rd period of thereafter to the day of zero newly confirmed COVID-19 cases report (24 January to 27 March 2020); and (iv) 4th period of thereafter to the day when public traffic transportations reopened (28 March to 11 April 2020). Family function was regarded as an important independent variable in relation to PPD and was examined using the APGAR scale, which was developed by Smilkstein and determined family function from five dimensions (adaptation, partnership, growth, affection and resolution) [18]. Each dimension was divided into three levels with scores from 0 (hardly ever) to 2 (almost always). A total score of 0–3, 4–6 and 7–10 are designated as poor, fair and good family functions, respectively.

### Covariates

Demographics used as covariates included age (< 29, 30–34 and > 34 years), ethnicity (Han and others), education (junior high or below, senior high and college or more), urban/rural, family income of last year (< 50,000 RMB, 50,000–100,000 RMB, ≥100,000 RMB and unclear), insurance (yes or no) and first-time father (yes or no). Other covariates of health behaviour factors, including smoking/passive smoking within 1 year (yes or no) and exercise within 1 year (yes or no), were also examined.

### Data analysis

All statistical analyses were performed using SAS 9.4 for Windows. Chi-square test was used to analyse the between-group differences of depression prevalence stratified by factors as the COVID-19 pandemic periods, family function, demographics (age, ethnicity, education, urban/rural, family income, insurance and first-time father), and health behaviour factors (smoking and exercise). Multiple logistic regression modelling was applied to analyse the dependency of prevalent depression on the mentioned explanatory variables. Model 1 included the periods of the COVID-19 pandemic and family function as independent variables. Model 2 analysed the associations of the COVID-19 pandemic, family function and depression risk in the adjustment of demographics. Model 3 involved health behaviour factors for covariate adjustment in addition to those in model 2. Results from multiple logistic regression analyses were reported as adjusted odds ratios (OR) and 95% confidence intervals (CI). All tests were two-sided, and a *p*-value of less than 0.05 was considered as statistically significant.

## Results

A total of 1341 participants were found eligible for this study. After the removal of individuals with incomplete questionnaires (*n* = 121) and patients with self-reported depression (*n* = 33), 1187 participants (88.52%) were finally included in the statistical analysis.

### General characteristics of participants

The prevalence of PPD was 13.82% throughout the COVID-19 pandemic (Table 1). Among the participants, 44.65% were aged 30–34 years, most (97.56%) were of Han nationality and the majority (77.25%) finished education in a college or beyond. The participants educated with junior high or below had a relatively high proportion of PPD (22.22%). More than half (68.13%) of the participants lived in urban cities, and 51.90% had a family income of more than 100,000 RMB. Compared with the fathers with family income ≥100,000 RMB, those with family income < 50,000 RMB had higher proportion of PPD (24.20% vs 11.36%, *p* < 0.01). Approximately 93.43% of fathers had insurance, and those without insurance had a relatively higher proportion of PPD (28.21% vs 12.80%, *p* < 0.01). Almost two-thirds of the participants (67.31%) were first-time fathers and had significantly lower proportion of PPD than the others (11.83% vs 18.41%, *p* < 0.01).

**Table 1:** General characteristics of involved participants in this study

Variables	No depression N (%)	Depression N (%)	Total N (%)	***χ***^***2***^	***P***
**Paternal characteristics**					
Total	1023 (86.18)	164 (13.82)	1187 (100.00)		
Age				7.12	0.03
< 29	309 (89.57)	36 (10.43)	345 (29.06)		
30–34	457 (86.23)	73 (13.77)	530 (44.65)		
> 34	257 (82.37)	55 (17.63)	312 (26.28)		
Ethnicity				2.68	0.10
Han	995 (85.92)	163 (14.08)	1158 (97.56)		
Other	28 (96.55)	1 (3.45)	29 (2.44)		
Education				8.62	0.01
Junior high or below	63 (77.78)	18 (22.22)	81 (6.82)		
Senior high	156 (82.54)	33 (17.46)	189 (15.92)		
College or beyond	804 (87.68)	113 (12.32)	917 (77.25)		
Urban/rural				3.30	0.07
Rural	316 (83.60)	62 (16.40)	378 (31.87)		
Urban	707 (87.50)	101 (12.50)	808 (68.13)		
Family income (RMB)				18.29	< 0.01
< 50,000	119 (75.80)	38 (24.20)	157 (13.23)		
50,000-100,000	259 (85.48)	44 (14.52)	303 (25.53)		
≥ 100,000	546 (88.64)	70 (11.36)	616 (51.90)		
Unclear	99 (89.19)	12 (10.81)	111 (9.35)		
Insurance				14.50	< 0.01
Yes	967 (87.20)	142 (12.80)	1109 (93.43)		
No	56 (71.79)	22 (28.21)	78 (6.57)		
First-time father				8.56	< 0.01
Yes	641 (88.17)	86 (11.83)	727 (67.31)		
No	288 (81.59)	65 (18.41)	353 (32.69)		
**Health-related behaviors**					
Smoking				14.06	< 0.01
No	609 (89.30)	73 (10.70)	682 (58.84)		
Yes	389 (81.55)	88 (18.45)	477 (41.16)		
Exercise				2.52	0.11
No	341 (83.99)	65 (16.01)	406 (35.06)		
Yes	657 (87.37)	95 (12.63)	752 (64.94)		

No depression: EPDS score 0–9; Depression: EPDS score 10–30

Less than half of the participants (41.16%) reported smoking during the past year and had higher proportion of PPD than those who did not smoke (18.45% vs 10.70%, *p* < 0.01). Approximately 64.94% of participants had a habit of exercise in the last year.

### Independent variables

**Fig. 1:**
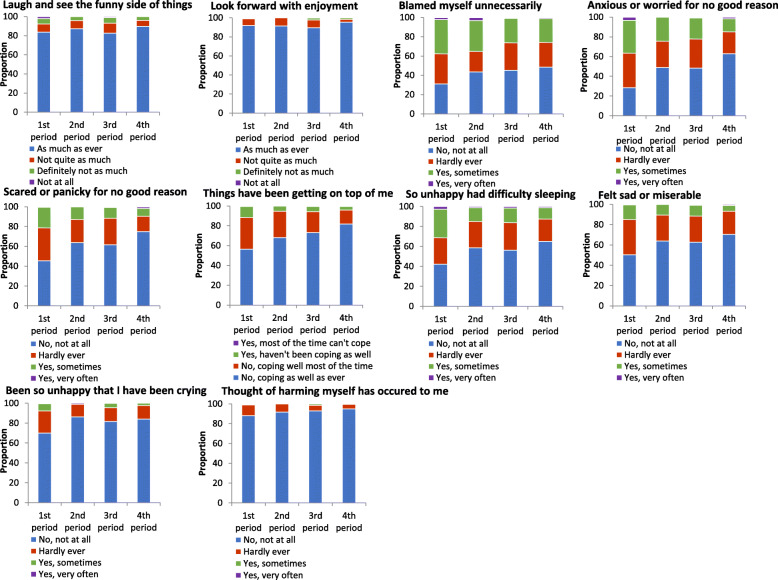
Response for ten Edinburgh postnatal depression scale questions during COVID-19 pandemic. 1st period: before the announcement of human-to-human transmission (31 December 2019 to 18 January 2020); 2nd period: from the announcement of human-to-human transmission to traffic restrictions (19–23 January 2020); 3rd period: Traffic restrictions (24 January 2020 to 27 March 2020); 4th period: Traffic restrictions dismissed (28 March 2020 to 11 April 2020)

Table 2 and Fig. 1 show the prevalence of PPD during the four periods of the COVID-19 pandemic (31 December 2019 to 11 April 2020). The mean score of EPDS in the first period was 5.97 and the prevalence of PPD in the first period was significantly higher than that in the other three periods (19 January 2020 to 11 April 2020). Moreover, the majority of fathers (86.18%) reported good family function and had lower PPD than those who reported fair family function (11.53% vs 32.31%, *p* < 0.01).

**Table 2:** Descriptive statistics for independent variables of participants during the COVID-19 pandemic

Variables	Mean (SD)	No depression N (%)	Depression N (%)	Total N (%)	***χ***^***2***^	***P***
Period of COVID-19 pandemic^a^					16.25	< 0.01
1st period	5.97 (4.89)	143 (78.14)	40 (21.86)	183 (15.42)		
2nd period	4.11 (4.12)	80 (85.11)	14 (14.89)	94 (7.92)		
3rd period	4.22 (4.59)	638 (86.80)	97 (13.20)	735 (61.92)		
4th period	3.22 (4.29)	162 (92.57)	13 (7.43)	175 (14.74)		
Family function					41.89	< 0.01
Poor	2.50 (0.71)	30 (88.24)	4 (11.76)	34 (2.86)		
Fair	5.37 (0.56)	88 (67.69)	42 (32.31)	130 (10.95)		
Good	9.48 (0.86)	905 (88.47)	118 (11.53)	1023 (86.18)		

No depression: EPDS score 0–9; Depression: EPDS score 10–30

^a^1st period: before the announcement of human-to-human transmission (31 December 2019 to 18 January 2020); 2nd period: from the announcement of human-to-human transmission to traffic restrictions (19–23 January 2020); 3rd period: Traffic restrictions (24 January 2020 to 27 March 2020); 4th period: Traffic restrictions dismissed (28 March 2020 to 11 April 2020)

### Predictors of PPD

The participants had significantly lower PPD risk in the 3nd (OR = 0.54, 95% CI: 0.34, 0.86) and 4rd (OR = 0.29, 95% CI: 0.14, 0.59) periods of the COVID-19 pandemic than in the 1st period (Table 3). Poor/fair family function was significantly associated with elevated PPD risk (OR = 2.93, 95% CI: 1.90, 4.52). The participants with low family income (< 50,000 RMB) had higher PPD risk than those with RMB 50,000–100,000 family income (OR = 1.91, 95% CI: 1.15, 3.20). Smoking men were more likely to suffer PPD than those who did not smoke (OR = 1.65, 95% CI: 1.15, 2.35).

**Table 3:** Logistic regression analysis for the effects of independent variables on paternal perinatal depression

Variables	Crude OR (95% CI)	Adjusted OR (95% CI)	Adjusted OR (95% CI)
Period of COVID-19 pandemic (in reference to 1st period)^a^			
2nd period	0.68 (0.34, 1.34)	0.65 (0.32, 1.32)	0.64 (0.31, 1.31)
3rd period	0.60 (0.39, 0.94)^*^	0.54 (0.34, 0.85)^**^	0.54 (0.34, 0.86)^**^
4th period	0.32 (0.16, 0.63)^**^	0.29 (0.14, 0.59)^**^	0.29 (0.14, 0.59)^**^
Family function			
Poor/fair vs Good	3.20 (2.12, 4.83)^**^	3.02 (1.97, 4.63)^**^	2.93 (1.90, 4.52)^**^
Age (in reference to < 29 years)			
30–34		1.41 (0.89, 2.23)	1.42 (0.90, 2.26)
> 34		1.78 (1.06, 3.00)^*^	1.74 (1.03, 2.94)^*^
Education (in reference to Junior high or below)			
Senior high		0.90 (0.45, 1.82)	0.92 (0.46, 1.87)
College or more		0.80 (0.41, 1.57)	0.86 (0.44, 1.70)
Urban/rural			
Urban vs Rural		0.87 (0.58, 1.32)	0.88 (0.58, 1.32)
Family income (in reference to ≥100,000 RMB)			
50,000-100,000		1.17 (0.75, 1.83)	1.11 (0.71, 1.73)
< 50,000		2.02 (1.21, 3.36)^**^	1.91 (1.15, 3.20)^*^
Insurance			
No vs Yes		2.22 (1.25, 3.94)^**^	2.29 (1.28, 4.10)^**^
First-time father			
No vs Yes		1.36 (0.90, 2.04)	1.33 (0.88, 2.00)
Smoking			
Yes vs No			1.65 (1.15, 2.35)^**^
Exercise			
Yes vs No			0.83 (0.58, 1.20)

^**^p < 0.01, ^*^p < 0.05

^a^1st period: before the announcement of human-to-human transmission (31 December 2019 to 18 January 2020); 2nd period: from the announcement of human-to-human transmission to traffic restrictions (19–23 January 2020); 3rd period: Traffic restrictions (24 January 2020 to 27 March 2020); 4th period: Traffic restrictions dismissed (28 March 2020 to 11 April 2020)

## Discussion

This is a unique study evaluated the depression risk of fathers in relation to partner delivery during the COVID-19 pandemic in Wuhan, China. The prevalence of PPD was 13.82%, which is in line with a systematic review stating that the prevalence of PPD evaluated by EPDS (scores > 9) was 3.00–30.50% [19] but is higher than that reported by Rao et al. (10.12% in the third trimester and 8.98% in 1 month of postnatal) [20]. Various cultural factors, different assessment tools of depression and inconsistent perinatal period cut-off may result in the differences in the distinct prevalence of PPD. Consistent with previously reports, the current study showed that the occurrence of PPD was 11.83% among first-time fathers, which was higher than that among second-time fathers (18.41%) [21]. This phenomenon could be related to the high parenting responsibilities of second-time fathers.

Critically, our study revealed that the prevalence of PPD peaked to the highest before the human-to-human transmission announcement in the COVID-19 pandemic, and the traffic restriction led to a reduced depression risk. A similar result was observed among women who delivered at the Soroka University Medical Center [22]. The high risk of PPD early in the pandemic is attributed to the public concern of the obscure and uncertainty of the pandemic. As an effective measure against COVID-19 spread, traffic restriction might have helped in reducing PPD with a decreased likelihood of viral infection in their partners and babies. This finding indicated that effective prevention and control measures against the pandemic successfully aided in alleviating PPD.

Similar results were observed for the 10 items of EPDS. Specifically, fathers with thoughts of self-harm were at a high risk of self-harm behaviours and suicide. To the best of our knowledge, this study is the first to report the decreased prevalence of self-harm ideation in fathers during the quarantine period. A previous work concentrated on the depression of pregnant women who showed high risks of depression and self-harm ideation during the COVID-19 outbreak in China [23]. Another systematic review failed to provide evidence on the increased self-harm ideation among general population during the COVID-19 pandemic [24]. This bias might be due to biological and psychosocial mechanisms of women who could be psychosocially vulnerable and have higher risk of self-harm ideation than men, especially for those in their perinatal period [25].

Family dysfunction is an important determinant of depression risks among adolescents [26], students [27], caregivers and patients [28] and the elderly [29]. Women’s satisfaction with family function is a protective factor against mental health disturbance [30]. This study further supported the observation that fathers who reported poor or fair family function had a high depression risk. Family function also served as a strong predictor of PPD (OR = 2.93). Wu et al. confirmed that family support is the most relevant factor of perinatal depression during the COVID-19 pandemic [23]. Measures against the COVID-19 pandemic including traffic restrictions and home quarantine forced fathers to stay with their family for longer time than usual. This situation could emphasize the importance of family function in the development of PPD during the COVID-19 pandemic. Future longitudinal studies are necessary to investigate the causal relationship between family function and PPD risk.

As another determinant of paternal depression, low household income contributed to increased depression risk among new fathers. This finding was in line with previous studies [31, 32]. The arrival of a new baby aggregates the financial pressure on new fathers. However, a relatively low number of fathers reported poor family income. This factor is dynamic, and the income of maternal partners during pregnancy is often reduced compared with that during the pre-pregnancy period. With the improvement of family income, the PPD risk may be considerably reduced.

Maternal depression is related to smoking before and within pregnancy [33–35]. In this study, fathers with active and passive smoking behaviours showed an elevated depression risk. This phenomenon could be partly explained by the neurobiological impact of nicotine intake through smoking on the brain part in charge of depression mood [36]. Patients with paternal depression could develop a habit of smoking, thus leading to the positive association between smoking and PPD.

This study has several limitations. Firstly, the cross-sectional design limited the causal relationship observation among factors. Secondly, data were self-reported, thereby possibly compromising the measurement accuracy of PPD and other related factors. Thirdly, the findings were only focused on targeted fathers in hospitals and cannot be generalized to those outside hospitals within the entire perinatal period. Fourthly, whether the women were in their prenatal or postnatal period when fathers participated in the survey has not been recorded. Moreover, this study drew conclusions based on findings from one hospital in Wuhan. Multi-centre studies are necessary to further address the issues related to PPD.

## Conclusions

Depression in perinatal mothers has been widely studied, but that in fathers is underestimated. The mental health of mothers and fathers must be promoted. This work presented a declined risk of PPD over the traffic restriction period. An improved family function may help alleviate the risk of PPD during the pandemic. Household income and smoking were also found to be associated with PPD risk. These findings provide evidence for health authorities to formulate targeted prevention and control strategies against PPD in relation to partner delivery during the COVID-19 pandemic.

## Supplementary Information

**Additional file 1.**

## Data Availability

The dataset used and/or analyzed during the current study are available from the corresponding author on reasonable request.

## Abbreviations

COVID-19: Coronavirus Disease 2019
EPDS: Edinburgh Postnatal Depression Scale
OR: Odds Ratios
CI: Confidence Intervals
PPD: Paternal perinatal depression
