# Supplementary material for: Risk assessment of paternal depression in relation to partner delivery during COVID-19 pandemic in Wuhan, China
Source: BMC Psychiatry. 2021 Jul 2;21:327. doi: 10.1186/s12888-021-03325-9 (PMC8253468; doi:10.1186/s12888-021-03325-9)
Supplement: Supplementary file 1 — Additional file 1. [file 12888_2021_3325_MOESM1_ESM.docx]

Dear fathers in hospital, in order to understand your mental health status and medical service demands, the Obstetrics Department of Hubei maternal and child health hospital developed this survey. The participation of the survey is voluntary, anonymous and confidential. The results of the survey will not be used for other purposes other than scientific research. Thank you for your participation!

1. Date (Automatically obtained from the Questionnaire star): ____________
2. What's your age? ____ years
3. What's your ethnicity?

①Han ②Other

1. What’s your highest education?

①Junior high or below ②Senior high ③College or beyond

1. Are you resident in urban or rural areas?

①Urban ②Rural

1. What was the total income of your family last year?

①<50,000 RMB ②50,000-100,000 RMB ③≥100,000 RMB ④Unclear

1. Do you currently have medical insurance (such as the basic medical insurance for urban residents, the basic medical insurance for urban workers, the New rural cooperative medical system, commercial medical insurance, et al.)

①Yes ②No

1. Is this the first time to be a father?

①Yes ②No

1. Do you have the habit of smoking or passive smoking in the past year?

①Yes ②No

1. Do you have the habit of exercise in the past year (Each time lasts more than 20 minutes)?

①Yes ②No

1. Are you satisfied that you can turn to your family for help when something is troubling you?

①Almost always ②Some of the time ③Hardly ever

1. Are you satisfied with the way your family talks over things with you and shares problems with you?

①Almost always ②Some of the time ③Hardly ever

1. Are you satisfied that your family accepts and supports your wishes to take on new activities or directions?

①Almost always ②Some of the time ③Hardly ever

1. Are you satisfied with the way your family expresses affection and responds to your emotions, such as anger, sorrow, and love?

①Almost always ②Some of the time ③Hardly ever

1. Are you satisfied with the way your family share time with you together?

①Almost always ②Some of the time ③Hardly ever

1. Have you been able to laugh and see the funny side of things during the past seven days?

①As much as ever ②Not quite as much ③Definitely not as much ④Not at all

1. Have you looked forward with enjoyment to things during the past seven days?

①As much as ever ②not quite as much ③definitely not as much ④not at all

1. Have you blamed yourself during the past seven days?

①No, not at all ②Hardly ever ③Yes, sometimes ④Yes, very often

1. Have you been anxious or worried for no good reasons during the past seven days?

①No, not at all ②Hardly ever ③Yes, sometimes ④Yes, very often

1. Have you felt scared or panicky for no very good reason during the past seven days?

①No, not at all ②Hardly ever ③Yes, sometimes ④Yes, very often

1. Have you felt things have been getting on top during the past seven days?

①No, coping as well as ever ②No, coping well most of the time

③Yes, haven’t been coping as well ④Yes, most of the time can’t cope

1. Have you felt been so unhappy that you have had difficulty sleeping during the past seven days?

①No, not at all ②Hardly ever ③Yes, sometimes ④Yes, very often

1. Have you felt sad or miserable during the past seven days?

①No, not at all ②Hardly ever ③Yes, sometimes ④Yes, very often

1. Have you been so unhappy that you have been crying during the past seven days?

①No, not at all ②Hardly ever ③Yes, sometimes ④Yes, very often

1. Does the thought of harming yourself has occurred to you during the past seven days?

①No, not at all ②Hardly ever ③Yes, sometimes ④Yes, very often
